# Differences in Diet and Gut Microbiota Between Lactating and Non-lactating Asian Particolored Bats (*Vespertilio sinensis*): Implication for a Connection Between Diet and Gut Microbiota

**DOI:** 10.3389/fmicb.2021.735122

**Published:** 2021-10-12

**Authors:** Jingjing Li, Yujia Chu, Wenwen Yao, Hui Wu, Jiang Feng

**Affiliations:** ^1^College of Life Science, Jilin Agricultural University, Changchun, China; ^2^Jilin Provincial Key Laboratory of Animal Resource Conservation and Utilization, Northeast Normal University, Changchun, China

**Keywords:** diets, gut microbiota, lactation, bats, composition

## Abstract

In mammals, lactation is considered the most energetically costly phase for females. To meet nutritional and energy demands, lactating females usually change feeding patterns by eating food that is higher in protein and calories. Their gut microbes respond accordingly to help adapt to the changes in diet. In this study, we examined differences in diet and gut microbial composition between lactating and non-lactating Asian particolored bats (*Vespertilio sinensis*) using COI and 16S amplicon sequencing. When compared with non-lactating bats, we found that the diversity and composition of lactating bats’ diets differed; the proportion of Diptera increased and Coleoptera and Orthoptera decreased significantly. This could be attributed to the easy availability and high protein content of Diptera. Comparative analysis of the gut microbiota of lactating and non-lactating females showed that although the diversity of gut microbiota did not change, the relative abundance of specific gut microbiota associated with a particular diet did change. For example, when the consumption of Coleoptera decreased in lactating bats, the relative abundance of Lactobacillaceae was also reduced. Lactobacillaceae are thought to be involved in the digestion of Coleopteran exoskeletons. This study suggests that during lactation, Asian particolored bats eat a diet that yields higher levels of protein, and at the same time, the abundance of specific gut microbes change to help their hosts adapt to these changes in diet.

## Introduction

In the animal kingdom, females typically play a larger role in the care of young offspring. To maximize the chances of their offspring’s survival, they invest more energy and pay a higher parental cost by providing larger sex cells and they invest more energy into rearing them (Taylor et al., 2000). This phenomenon is most prominent in lactating female mammals. For example, in a study of lemurs, the females’ energy expenditure increased as their babies grew (Tarnaud, 2006). Similarly, in non-human primates, females showed higher dietary protein requirements during lactation (Oftedal, 1991). Therefore, lactation is the most costly investment in offspring by female mammals. To meet their infants’ needs for energy and nutrition, females face greater energetic and nutritional pressure during lactation (Aiello and Wells, 2002). This is especially true for the only flying mammals, bats. During reproduction, males and females bats of many species inhabit separate locations and females form separate breeding colonies to carry out the young offspring duties alone (Rydell and Baagøe, 1994; Safi et al., 2007). And during lactation, females require the high energy for milk production as well as for carrying their young while flying (Kunz, 1987; Sigsgaard et al., 2020).

Optimal foraging theory (OFT) suggests that an animal will attempt to gain the greatest energetic benefit for the lowest energetic cost, while foraging to maximize fitness (Schoener, 1971). Although, an individual can consume a wide diversity of prey items, it may adopt different diets and select specific foods depending on energetic benefits relative to handling time costs (Araujo et al., 2011). On the basis of the energetic demands of lactation in previous studies, lactating females take in more energy than females in other reproductive states (Herrera and Heymann, 2004; Wichert et al., 2009). They may accomplish this by eating for longer (Denryter et al., 2020), eating faster (McCabe and Fedigan, 2007), eating more nutritious food (Herrera and Heymann, 2004) or all three. Dietary changes are one of the most effective ways to cope with these energy and nutrition needs (Denryter et al., 2020). Many studies have emphasized that selection of high-quality foods, rather than bulk feeding, is the most important component of nutrient acquisition during lactation (Vasey, 2000, 2002). State-dependent diets, especially reproductive state-dependent diets, have been demonstrated in many mammal species (Thompson and Wrangham, 2008). To our knowledge, no study has yet examined whether the composition of lactating bats’ diets differs from that of non-lactating bats, and whether lactating bats choose to eat more high-protein foods in response to higher energy and nutritional requirements.

The gut microbiota is the most abundant microbial system co-occurring with mammals and is largely a consequence of both the nutrient-rich environment of mammalian digestive systems and the beneficial functions these communities provide to their hosts (Muegge et al., 2011). In mammals, the gut microbiota is expected to adapt to the varying energetic and nutritional pressures of females in different reproductive states. Recent studies implied that the community composition and diversity of the gut microbiota can covary with dietary changes, and that these changes are beneficial to the host, such that they can meet the energy and nutrition requirements of lactation (Phillips et al., 2017). So far, most research on lactating females has focused on the effects of the altered reproductive status and hormones on gut microbiota (Mallott et al., 2020; Sun et al., 2020); few studies have examined whether changes in diet composition during lactation cause corresponding changes in gut microbiota. To examine the food composition and gut microbial structure of mammals during lactation, and try to establish the relationship between diet and gut microbiota during their most energetically costly phase, we focused on an insectivorous bat species in northeast China: the Asian particolored bat (*Vespertilio sinensis*). In this study, COI and 16S rRNA amplicon sequencing was used to investigate the diet and gut microbiota of adult females in and out of lactation periods. We hypothesized that lactating bats, compared with non-lactating bats, change their diet composition to meet higher energy and nutritional requirements and their gut microbial diversity and composition will change accordingly. We predict that (1) there are significant differences in the composition of diet between lactating and non-lactating bats; (2) gut microbial diversity and composition of lactating bats are different from that in non-lactating bats; and (3) there is a strong correlation between diet and gut microbes.

## Materials and Methods

### Sample Collection

To avoid the influence of age and season (Ma et al., 2007), all female bats in this study were randomly collected from July to August 2020 in the Asian particolored bats habitat under the highway bridge of Acheng District, Harbin City, Heilongjiang Province, China (45°55'N, 126°94'E). Asian particolored bats mainly feed on insects and inhabit the roofs or eaves of bridges or old buildings. During reproduction, males and females inhabit separate locations, which provides ideal conditions for sampling. Bats were captured with a mist net as they returned to their habitat after hunting. We gently caught the bats from the net by hand with sterile gloves. We released the bats from the net as soon as possible and none were injured. We collected lactating (*n*=10) and non-lactating (*n*=14) Asian particolored bats in two periods. The determination of reproductive status was based on external morphological characteristics: the nipples of female bats become enlarged during lactation and the hairs around the nipples of female bats disappear because of suckling by their infants (Haarsma, 2008; Racey, 2009). Each captured individual was put into sterilized kraft bags to collect their excrement. After defecation, fecal samples were immediately put into an empty cryopreservation tube with sterile forceps and placed into dry ice for preservation until they were transported to the laboratory, where they were frozen at −80°C until DNA extraction. On completion of the sampling, the bats were immediately released back to their habitat.

### DNA Extraction and PCR Amplification

FastDNA™ Spin Kit (Santa Ana, California, United States) was used to extract total insect and bacterial genomic DNA samples following the manufacturer’s instructions, and the extracted DNA was tested. The quantity of extracted DNA was measured using a NanoDrop 2000 spectrophotometer (Thermo Fisher Scientific, Waltham, MA, United States) and the quality was assessed using agarose gel electrophoresis.

Forward primer ZBJ-ArtF1cF and reverse primer ZBJ-ArtR2cR were used for Cytochrome oxidase fragments of PCR amplification, and forward primers 338F and reverse 806R were used for PCR amplification of the V3–V4 region of bacterial 16S rRNA gene. The PCR reaction system and conditions are described in detail in the Supplementary Material. PCR amplicons were purified with Agencourt AMPure Beads (Beckman Coulter, Indianapolis, IN, United States) and quantified using the PicoGreen dsDNA Assay Kit (Invitrogen, Carlsbad, CA, United States). After the individual quantification step, amplicons were pooled in equal amounts and pair-end 2×300-bp sequencing was performed using the Illlumina MiSeq platform with MiSeq Reagent Kit v3.

### Bioinformatics Analysis

Raw fastq files were quality-filtered by Trimmomatic (Bolger et al., 2014) and merged by FLASH (Magoč and Salzberg, 2011). After performing quality control to obtain the optimized sequences, the operational taxonomic units (OTUs) were clustered using Usearch (Edgar, 2010) with singletons removed and chimera filtering. All optimized sequences were mapped to representative sequences, and sequences with >97% similarity with the representative sequence were selected to generate an OTU table. All analyses of the sequencing filtering and normalization were performed in Usearch. Sequencing effort coverage was visually assessed using alpha diversity rarefaction curves of number of OTUs.

### Diet Data Analysis

Taxonomic identification of insect species was made by comparing a representative sequence from each OTU to reference sequences in the Barcode of Life Database (BOLD)^1^ and the Genbank database.^2^ By doing so, the insects in the bats’ diet could be identified to the species level. Sequence data analyses were mainly performed using QIIME v1.8.0 (Caporaso et al., 2010) and R packages (v3.5.1). OTU-level ranked abundance curves were generated to compare the richness and evenness of OTUs among samples. The Shannon diversity index and Chao1 richness index were calculated using the OTU table in QIIME to determine the diet alpha diversity for the groups of bats. We then used the Wilcoxon rank-sum test to calculate significant differences. Beta diversity was analyzed to investigate variation in the dietary composition of the different group samples using unweighted UniFrac distance metrics. Differences in group dispersion among bat groups were assessed using ADONIS, using R package, vegan (Jari Oksanen et al., 2019). Beta diversity was then visualized with principal coordinate analysis (PCoA; Ramette, 2007). The species composition and relative abundance of bat diets in each group were calculated at order taxonomic levels, and the composition of dominant species in different groups was visualized by community Pie plots. The relative abundance of insect species at order, family, and genus consumed by lactating and non-lactating bats were analyzed using Wilcoxon rank-sum test in R package, stats (R Core Team, 2019).

### Gut Microbiota Data Analysis

QIIME software, Chao1 and Shannon indices were used to evaluate the alpha diversity of each sample. We then used the Wilcoxon rank-sum test to calculate significant differences between lactating and non-lactating bats. Beta diversity analysis was performed to investigate structural variation in microbial communities of the different group samples on the basis of unweighted UniFrac and weighted UniFrac distance metrics. Differences in group dispersion among bat groups were assessed using ADONIS. Beta diversity was then visualized with PCoA. Taxonomies were grouped at the phylum, family, and genus levels. Taxa abundances at the phylum, family, and genus levels were statistically compared among groups with using Wilcoxon rank-sum test, in R package, stats. To further identify the highly-dimensional gut microbes and characterize the differences between two or more biological conditions, we combined the linear discriminant analysis effect size (LEfSe) analysis.

### Diet and Gut Microbiota Correlation Analysis

We performed canonical correspondence analysis (CCA) to test the relative abundance of different insect species in the diet on microbial community at different taxonomic levels in lactating and non-lactating bats, applying forward selection and the Monte Carlo permutation test with 999 random permutations. In addition, we used Procrustes analysis to assess congruence between different insect species relative abundance dietary composition in different groups on microbial community structure and the significance of the Procrustes statistic was tested by 999 permutations with “protest” function.

## Results

### Diet

After quality processing, a total of 293,784 effective sequences were obtained, with an average effective sequence of 12,241 per sample. We identified 276 OTUs from reads, 99 of which were only found in the diet of lactating bats and 138 of which were only found in the diet of non-lactating bats (Supplementary Table 1).

**Figure 1:**
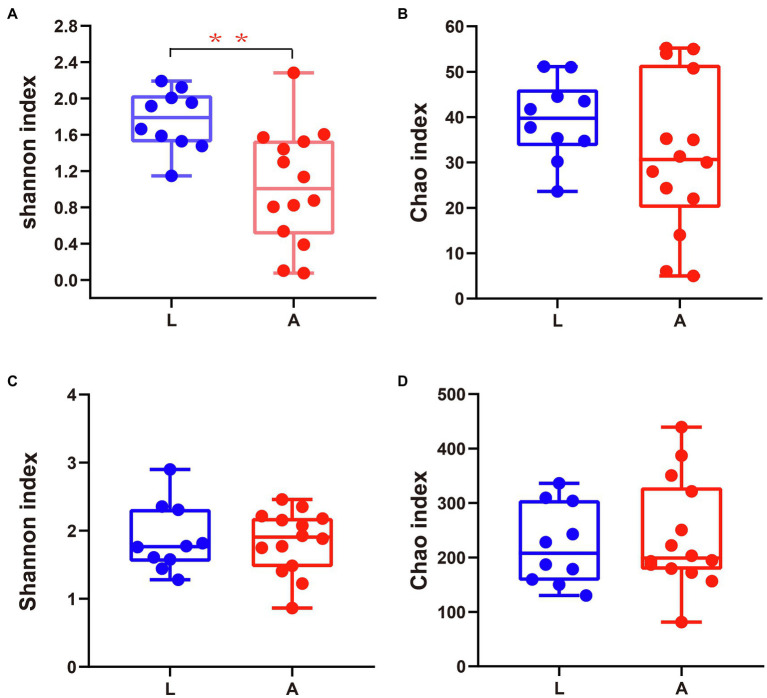
Alpha diversity of different groups. **(A,B)** The diet diversity estimated by Chao1 and Shannon indexes. **(C,D)** Gut microbial diversity estimated by Chao1 and Shannon indexes. L refers to lactating bats; A refers to non-lactating bats. ^*^0.01 < *p* ≤ 0.05, ^**^0.001 < *p* ≤ 0.01, and ^***^p ≤ 0.001.

**Figure 2:**
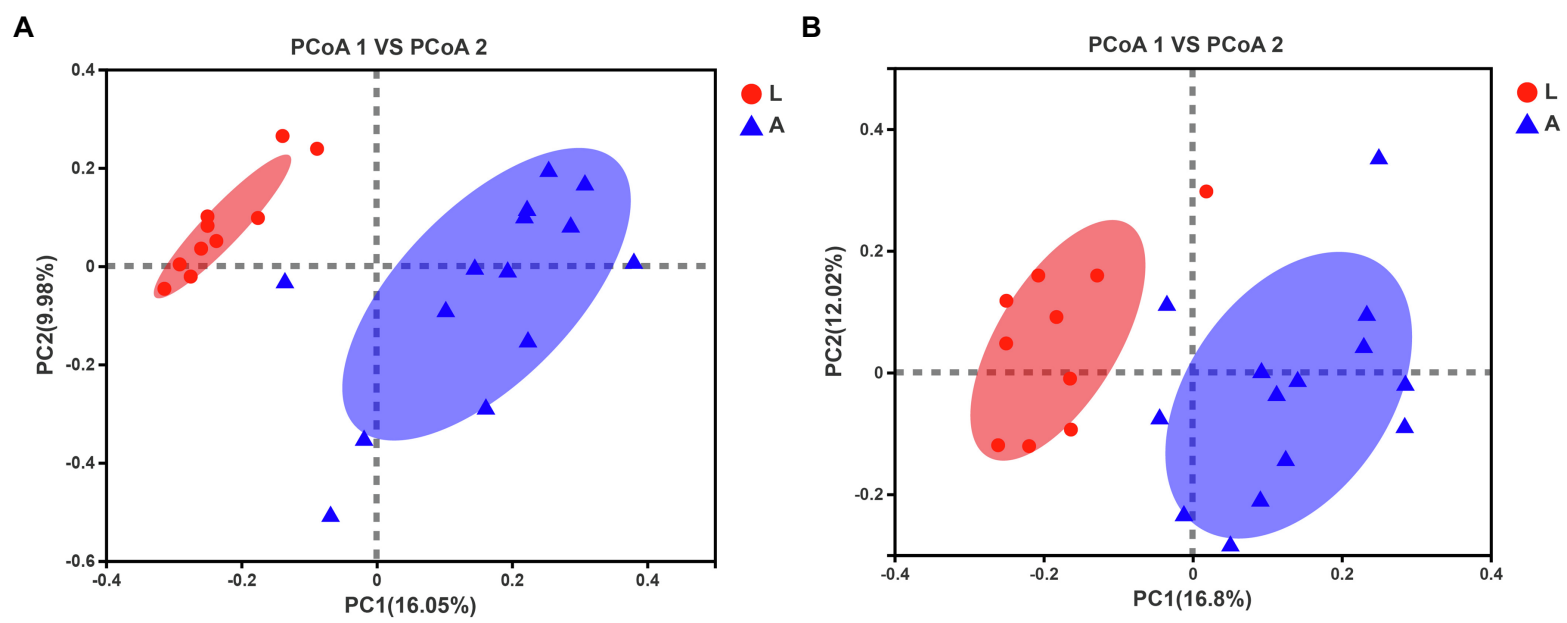
Principal coordinate analysis (PCoA) analysis based on unweighted UniFrac metrics. **(A)** Differential insect composition in diet between lactating and non-lactating bats. **(B)** Differential gut microbiota communities between lactating and non-lactating bats.

Dietary diversity differed significantly between the two reproductive periods. Compared with non-lactating bats, the alpha diversity of the diet based on the Shannon index was higher in lactating bats (Wilcoxon rank-sum test, *p*=0.004, *Q*=0.009; Figure 1A; Supplementary Figure 1A). However, the alpha diversity based on the Chao1 index was no different for lactating and non-lactating bats (Wilcoxon rank-sum test, *p*=0.23, *Q*=0.23; Figure 1B). The unweighted UniFrac metric data showed distinct clustering by lactating bat and non-lactating bat values, such that individuals from the two groups clustered separately. The results of ADONIS showed significant differences between the two groups (ADONIS: *R*^2^=0.14, *p*=0.001; Figure 2A).

**Figure 3:**
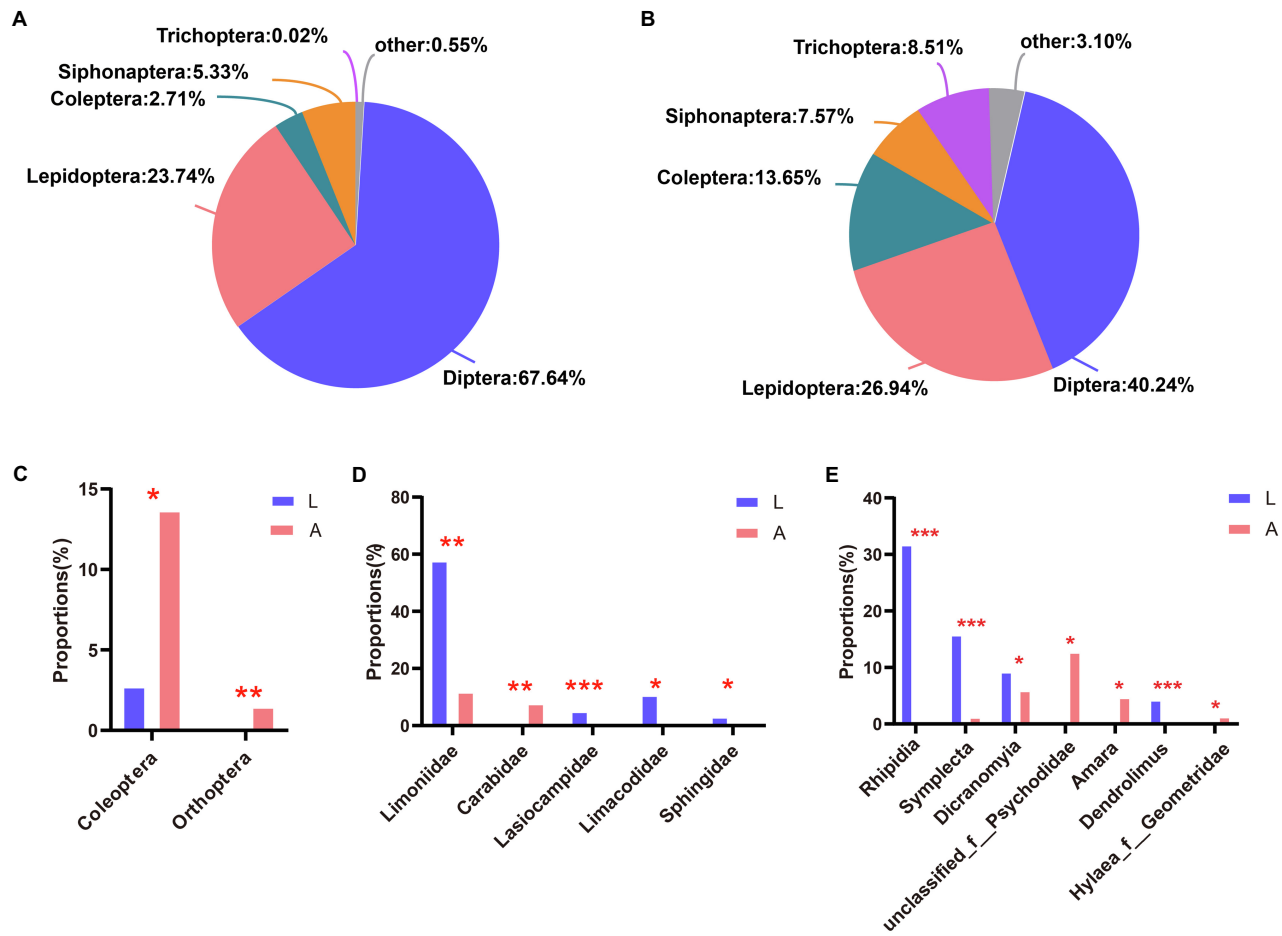
**(A,B)** Composition at the order level of insect species in the diet: **(A)** Lactating bats and **(B)** Non-lactating bats. **(C–E)** Analysis of insect species composition differences: **(C)** Analysis of order-level differences, **(D)** Analysis of family-level differences, and **(E)** Analysis of genus-level differences (^*^0.01<*p*≤0.05, ^**^0.001<*p*≤0.01, and ^***^p≤0.001).

Ten orders were found in the diet of lactating bats, with Diptera accounting for 67.64% and Lepidoptera for 23.74% (Figure 3A). Thirteen orders were found in the diet of non-lactating bats, with Diptera accounting for 40.24% and Lepidoptera for 26.94% (Figure 3B). We compared the relative read abundance of more than 1% of insect species and found that compared with non-lactating bats, lactating bats consumed more Diptera. The consumption of Coleoptera was significantly reduced (*p*=0.03), and the lactating bats even stopped consuming the larger Orthoptera (*p*=0.001; Figure 3C; Supplementary Table 3). At the family level, the proportion of Limoniidae (*p*=0.0002), Lasiocampidae (*p*=0.00002), and Limacodidae (*p*=0.04) were significantly increased and Carabidae (*p*=0.009) was significantly reduced in the diet of lactating bat diets (Figure 3D; Supplementary Table 3). At the genus level, the proportion of Rhipidia (*p*=0.000009), Symplecta (*p*=0.0002), Dicranomyia (*p*=0.02), and Dendrolimus (*p*=0.00003) were significantly increased and unclassified_f__Tipulidae (*p*=0.012), Amara (*p*=0.12), Hylaea_f__Geometridae (*p*=0.043; Figure 3E; Supplementary Table 3).

### Gut Microbiota

After quality inspection, 730,728 effective sequences were obtained (average of 30,447 reads/sample). We identified 995 OTUs from reads, 276 of which were only found in the gut microbiota of lactating bats and 380 of which were only found in the gut microbiota of non-lactating bats (Supplementary Table 1).

There was no significant difference in the alpha diversity of gut microbiotas between lactating bats and non-lactating bats (Wilcoxon rank-sum test, Chao1 index, *p*=0.62, *Q*=0.98, Shannon index, *p*=0.98, *Q*=0.98; Figures 1C,D; Supplementary Figure 1B). For the comparison of individual bat microbiotas, the PCoA results based on unweighted UniFrac metric data showed that individuals from the lactating and non-lactating groups clustered separately. The results of ADONIS showed significant differences between the two groups (ADONIS: *R*^2^=0.1387, *p*=0.001; Figure 2B). But weighted UniFrac measurements showed no difference between the lactating and non-lactating groups (ADONIS: *R*^2^=0.0755, *p*=0.137; Supplementary Figure 2).

**Figure 4:**
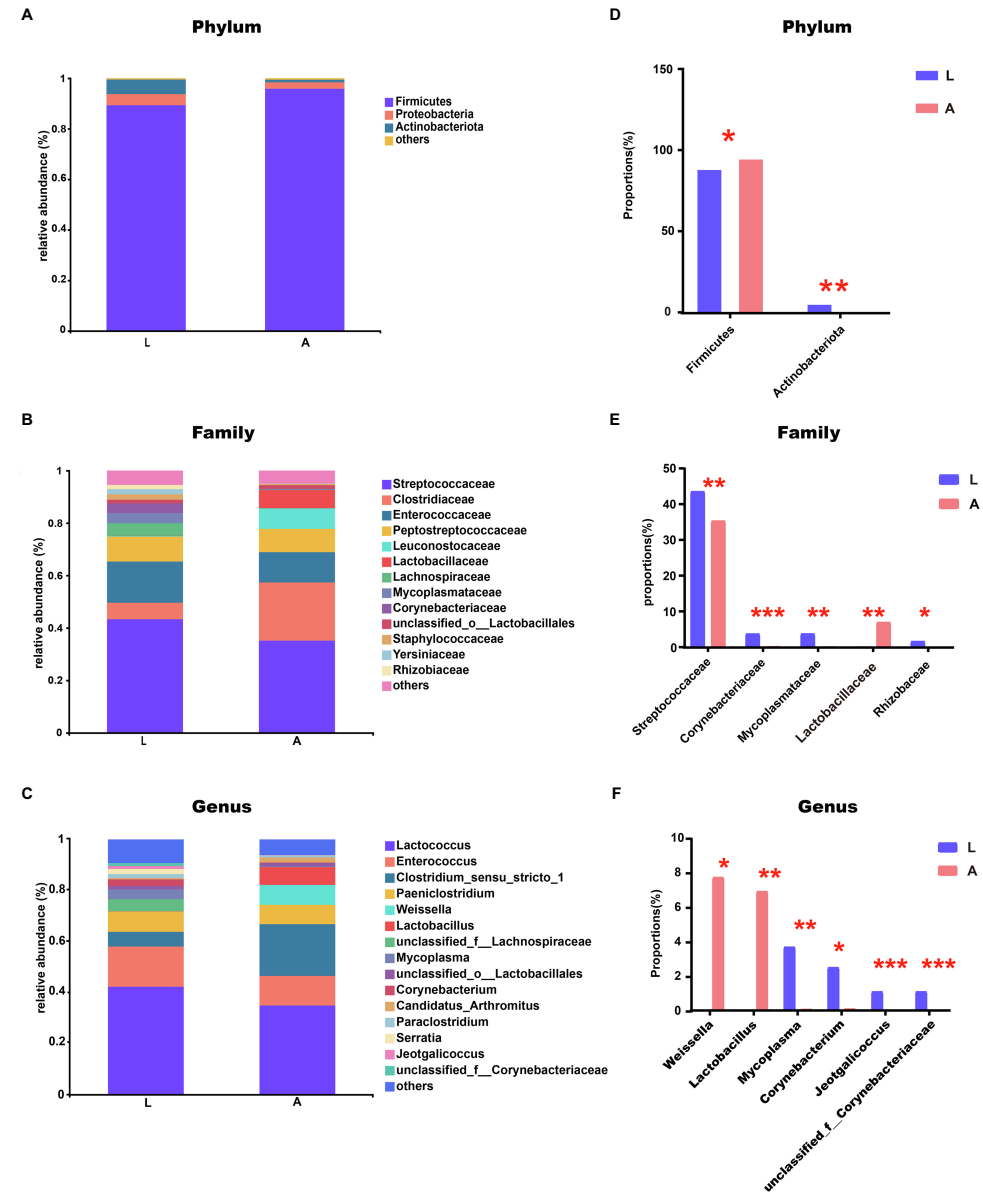
**(A–C)** Relative abundance of major taxa in microbiotas of the bat gut: **(A)** Phylum level, **(B)** Family level, and **(C)** Genus level. **(D–F)** Analysis of gut microbial community differences: **(D)** Analysis of phylum-level differences, **(E)** Analysis of family-level differences, and **(F)** Analysis of genus-level differences (^*^0.01<*p*≤0.05, ^**^0.001<*p*≤0.01, and ^***^p≤0.001).

Three major bacterial phyla with relative abundance greater than 1% were identified in Asian particolored bat microbiotas, and most sequences were classified as Firmicutes (93.14%), followed by Proteobacteria (3.32%) and Actinobacteria (3.02%; Figure 4A).

In terms of microbial composition, the relative abundances of some dominant microbial groups at the level of phylum, family, and genus changed significantly. At the phylum level, compared with the gut microbiotas of non-lactating bats, Actinobacteria significantly increased (*p*=0.004) and Firmicutes decreased (*p*=0.01) in lactating bats (Figures 4A,D; Supplementary Figure 3; Supplementary Table 3). At the family level, Corynebacteriaceae (*p*=0.0001), Staphylococcaceae (*p*=0.0001), Mycoplasmataceae (*p*=0.001), and Rhizobiaceae (*p*=0.02) significantly increased and Lactobacillaceae (*p*=0.001) significantly decreased in lactating bats (Figures 4B,E; Supplementary Figure 3; Supplementary Table 3). At the genus level, *Mycoplasma* (*p*=0.001), *Corynebacterium* (*p*=0.05), *Jeotgalicoccus* (*p*=0.0004), *unclassified_f__Corynebacteriaceae* (*p*=0.0001) significantly increased and *Weissella* (*p*=0.03) and *Lactobacillus* (*p*=0.001) significantly decreased in lactating bats (Figures 4C,F; Supplementary Figure 3; Supplementary Table 3).

### Correlation Between Diet and Gut Microbiota

**Figure 5:**
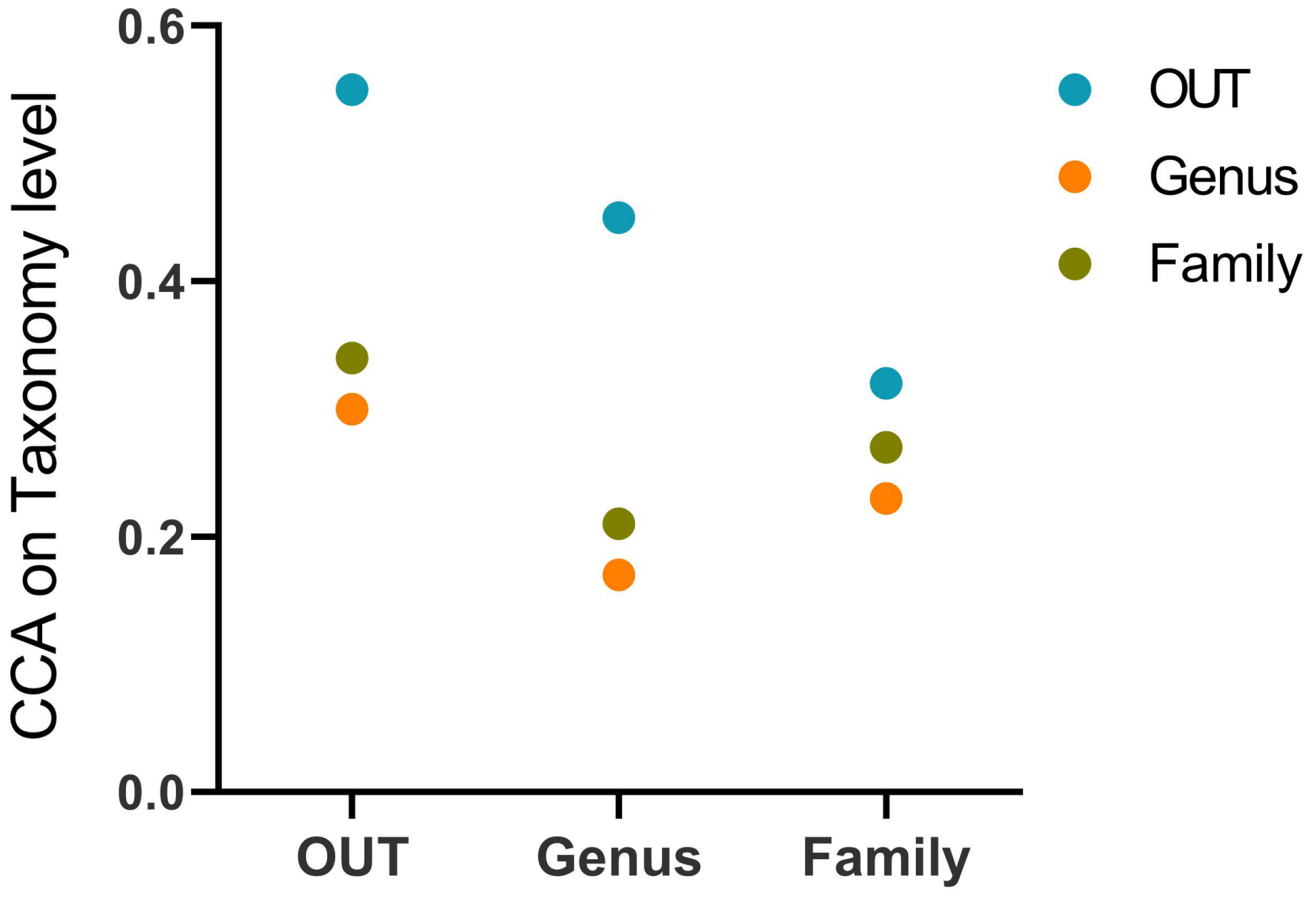
Correlations between the gut microbial datasets and the dietary datasets summarized at different taxonomic levels.

Because effects of dietary composition on gut microbiome community may vary with taxonomic level, we summarized gut microbiome community and dietary datasets at multiple taxonomic levels, and performed correlation score for each rank. CCA suggests that there was a significant correlation between diet and gut microbiota in lactating and non-lactating bats at OTU and family levels (*p*<0.05; Figure 5; Supplementary Table 4). At the genus level, diet was found to be only correlated with the OTU level of gut microbiome (*p*=0.004), but no significant correlations were observed between genus and family level of gut microbiome (*p*>0.05; Figure 5; Supplementary Table 4). The Procrustes analysis showed that there was a strong correlation between the diet and gut microbiome of lactating bats compared with non-lactating bats (*p*<0.05; Supplementary Table 5).

## Discussion

In this study, we examined the differences in diets and gut microbiotas between lactating and non-lactating bats *V. sinensis* and explored the correlations between the bats’ diets and the microbial composition of their guts during lactation and non-lactation periods. Our main findings are as follows: (1) Compared with non-lactating bats, the composition of lactating bats’ diets differed significantly, which suggests that lactating bats are selective in their diet. (2) Diversity of the gut microbiome did not differ between lactating and non-lactating bats, but relative abundance of gut microbes did vary. (3) Changes in the abundance of certain groups of gut microbes were associated with some particular types of foods, which confirms the role of diet in shaping gut microbiota. The results supported our hypothesis and prediction 1 and 3, while results comparing gut microbial diversity did not support our hypothesis and prediction 2.

### Diet Changes

Lactation is the most energetically costly activity during reproduction in mammalian females (Vanslambrouck et al., 2021). Bats are the only truly flying mammals and during lactation, in addition to the high energy expenditure required to produce milk, females have to carry their young while flying. This has been shown to require staggering energy expenditure (Kunz et al., 1995; McLean and Speakman, 1999). Our study suggests that to meet the high energy requirements of lactation, female *V. sinensis* have a significantly higher diversity of insect species in their diet than non-lactating females, and the proportion of specific groups in their diet changed significantly. For example, lactating bats relied heavily on Diptera and decreased their consumption of Orthoptera and Coleoptera. The consumption of Lasiocampidae and Limacodidae in lactating bats was also significantly higher than that in non-lactating bats. There are several reasons for these results. First, Diptera have a higher protein content than Lepidoptera (Churchward-Venne et al., 2017). Lactating bats increased their dietary diversity and chose high-protein foods to ensure their nutritional needs were met and that lactation functioned properly to provide nutrient factors that are essential for infant growth and development (Lee et al., 2016; Lee and Kelleher, 2016; Marangoni et al., 2016). Thus, it is evident that high-quality food selection, rather than bulk feeding, is the most important component of nutrient acquisition when nursing (Vesterinen et al., 2016). Second, OFT suggests that an animal will attempt to gain the greatest energetic benefit for the lowest energetic cost while foraging to maximize fitness. Coleoptera have large exoskeleton that take time to handle, so bats tend to eat less of them during lactation (Dufour and Sauther, 2002). Although Orthoptera has a higher protein content, most species are active during the day. Few nocturnal species are active on the ground, which makes them more difficult for bats to hunt (Whitby et al., 2020). Finally, lactating females consumed significantly more species of certain insect families of the order Lepidoptera than did non-lactating females. We speculated that these particular species may have higher quality proteins that are more effective in supplementing the nutritional and energetic needs of lactating females, but evidence for this is lacking. On the basis of these findings, the OFT is supported, such that during lactation, Asian particolored bats support their energy demands by eating more nutritious food, which increases their nutritional intake.

### The Gut Microbiota Changes

The gut microbiota plays an important role in the nutrition and health of the host (Al-Asmakh et al., 2014; Gentile and Weir, 2018). Dietary components have the capability to modulate the composition of this biota (Wu et al., 2011; Barrett et al., 2018). Although, we found differences in dietary composition between lactating and non-lactating bats, we did not detect significant differences in gut microbial diversity. The results suggest that healthy mammals tend to stabilize the diversity of their gut microbes in adulthood (Albenberg and Wu, 2014). Our results are consistent with studies in a variety of primates that confirmed that the alpha diversity of the gut microbial community remained stable throughout lactation. Studies on the human gut microbiota have shown that markers of microbial stability, such as richness and diversity, are often used as indicators of gut health (Gentile and Weir, 2018). In a few cases, they confirmed that the diversity of the intestinal microbiota of mammalian females changed with reproductive status (Gaona et al., 2019; Sun et al., 2020). However, these studies did not emphasize the role of dietary changes during lactation in shaping the structural diversity of gut microbiota (Phillips et al., 2012). On the contrary, they suggested that the diversity of gut microbiota in lactating females may be more susceptible to other factors besides diet, such as hormones and metabolism (Liu et al., 2019; Coleman et al., 2021).

Although, the overall diversity of gut microbes was unrelated to diet, we found that changes in diet had influences on specific, related microbiota. In human studies, it has been confirmed that dietary fat consumption is positively correlated with abundant Actinobacteria and negatively correlated with abundant Firmicutes (Abecia et al., 2007; Wu et al., 2011; Jin et al., 2021). Compared with non-lactating bats, the lactating bats had a higher relative abundance of Actinobacteria and lower relative abundance of Firmicutes, which would be useful for consuming more fat and reducing fat storage, thus improving the survival rate of offspring (Tarnaud, 2006; Hamady et al., 2008). *Lactobacillus* and *Weissella* mainly degrade macromolecules with complex structures to promote gut absorption (Famularo et al., 2005; Safika et al., 2019). Because of the reduced consumption of insects with large bodies and hard shells during lactation, it is speculated that the significant decrease in the relative abundance of *Lactobacillus* and *Weissella* in the intestines of *V. sinensis* during lactation is related to the decrease in the proportion of Coleoptera in the diet. *Lactobacillus* and *Weissella* participates in the production of short-chain fatty acids and maintains the balance of the gut and maintaining normal host health (Markowiak-Kopec and Slizewska, 2020). The relative abundance of Staphylococcaceae increased in lactating bats, which increased microflora nutrient acquisition by improving the hydrolysis of indigestible proteins and polysaccharides in the diets, and improving the energy efficiency of females to breastfeed their infants (Collado et al., 2008; Hunt et al., 2012; Rowland et al., 2018). In conclusion, by analyzing the correlation between diet and the gut microbiota at different classification levels, we confirmed that diet does have a regulatory effect on gut microbiota of lactating bats. Although there is no correlation between insect species at the genus level and gut microbes, this may be related to some unclassified species, but diet is only one of the factors regulating gut microbiota during lactation.

Mammals have evolved many behavioral and physiological strategies to meet the energy and nutrient requirements of their altered reproductive state. These strategies include eating longer, eating faster, eating more nutritious food, and enhancing metabolic efficiency (Bell and Bauman, 1997), as well as using seasonal reproduction (Bronson, 2009). In this study, we found that during lactation, Asian particolored bats did eat more nutritious food by consuming a diet that yields higher levels of protein. In addition, the abundance of specific microbes in their gut changed to help them adapt to changes to the composition of their diet. Because of limitations of sample size and the variables we considered, we were not able to fully reveal other behavioral and physiological changes caused by changes in mammalian reproductive status. Future studies should incorporate more factors to reveal the characteristics of adaptive evolution in different reproductive states of mammals, especially the adaptive mechanism of lactation, which is the reproductive period requiring the highest energy investments.

## Data Availability Statement

The datasets presented in this study can be found in online repositories. The names of the repository/repositories and accession number(s) can be found in the article/Supplementary Material.

## Ethics Statement

The animal study was reviewed and approved by the Laboratory Animal Welfare and Ethics Committee of Jilin Agricultural University.

## Author Contributions

JL and HW contributed to the design of the study. WY, and YC collected the feces samples in all period. JF and HW contributed to the writing of the manuscript. All authors contributed to the article and approved the submitted version.

## Funding

This work was supported by the National Natural Science Foundation of China (Grant Nos. 32071492 and 32171489) and the Jilin Provincial Natural Science Foundation (Grant No. 20200201186JC).

## Conflict of Interest

The authors declare that the research was conducted in the absence of any commercial or financial relationships that could be construed as a potential conflict of interest.

## Publisher’s Note

All claims expressed in this article are solely those of the authors and do not necessarily represent those of their affiliated organizations, or those of the publisher, the editors and the reviewers. Any product that may be evaluated in this article, or claim that may be made by its manufacturer, is not guaranteed or endorsed by the publisher.

## Footnotes

^1^ http://www.boldsystems.org/

^2^ http://www.ncbi.nlm.nih.gov/GenBank
