# Supplementary material for: Differences in Diet and Gut Microbiota Between Lactating and Non-lactating Asian Particolored Bats (Vespertilio sinensis): Implication for a Connection Between Diet and Gut Microbiota
Source: Front Microbiol. 2021 Oct 12;12:735122. doi: 10.3389/fmicb.2021.735122 (PMC8546350; doi:10.3389/fmicb.2021.735122)
Supplement: Supplementary file 1 [file Data_Sheet_1.docx]

Supplementary Material

# Supplementary Materials and Methods

PCR amplification of Cytochrome oxidase fragments

ZBJ-ArtF1cF primer: 5’- AGATATTGGAACWTTATATTTTATTTTTGG -3’

ZBJ-ArtR2cR primer: 5’- WACTAATCAATTWCCAAATCCTCC -3’

The reaction system is as follows:

2×NuHi HiFi MasterMix 10µl

Forward Primer (5 µM) 0.8µl

Reverse Primer (5 µM) 0.8µl

Template DNA 10ng/µl

H20 add to 20μl

Conditions for PCR amplification:

95℃ for 10 min

Denaturation at 95℃ for 30s

Annealing at 52℃ for 30 s

Extension at 72℃ for 60s

A final elongation step at 72℃for 10 min

10°C until halted by user

Followed by 45 cycles

PCR amplification of V3-V4 region of bacterial 16S rRNA gene

338F primers: 5’-ACTCCTACGGGAGGCAGCAG-3’

806R primers: 5’-GGACTACHVGGGTWTCTAAT -3’

The reaction system is as follows:

5×FastPfu Buffer 4µl

2.5 mM dNTPs 2µl

Forward Primer (5 µM) 0.8µl

Reverse Primer (5 µM) 0.8µl

FastPfu Polymerase 0.4µl

BSA 0.2µl

Template DNA 10ng

H20 add to 20μl

Conditions for PCR amplification:

95℃ for 3 min

Denaturation at 95℃ for 30s

Annealing at 53℃ for 30 s

Extension at 72℃ for 45 s

A final elongation step at 72℃for 10 min

10°C until halted by user

Followed by 29 cycles

# Supplementary Figures and Tables

## Supplementary Figures


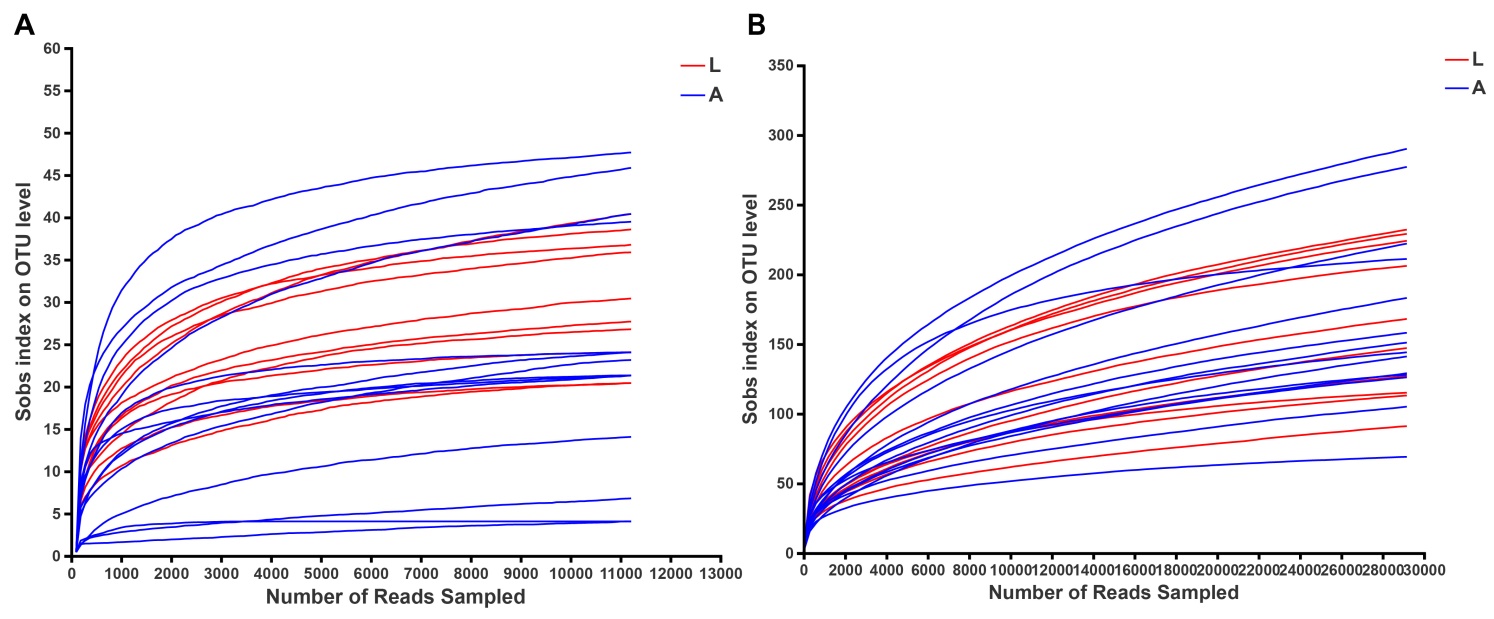


**Supplementary Figure 1.** Rarefaction curves: (a) rarefaction curves of diet (b) rarefaction curves of gut microbial communities.


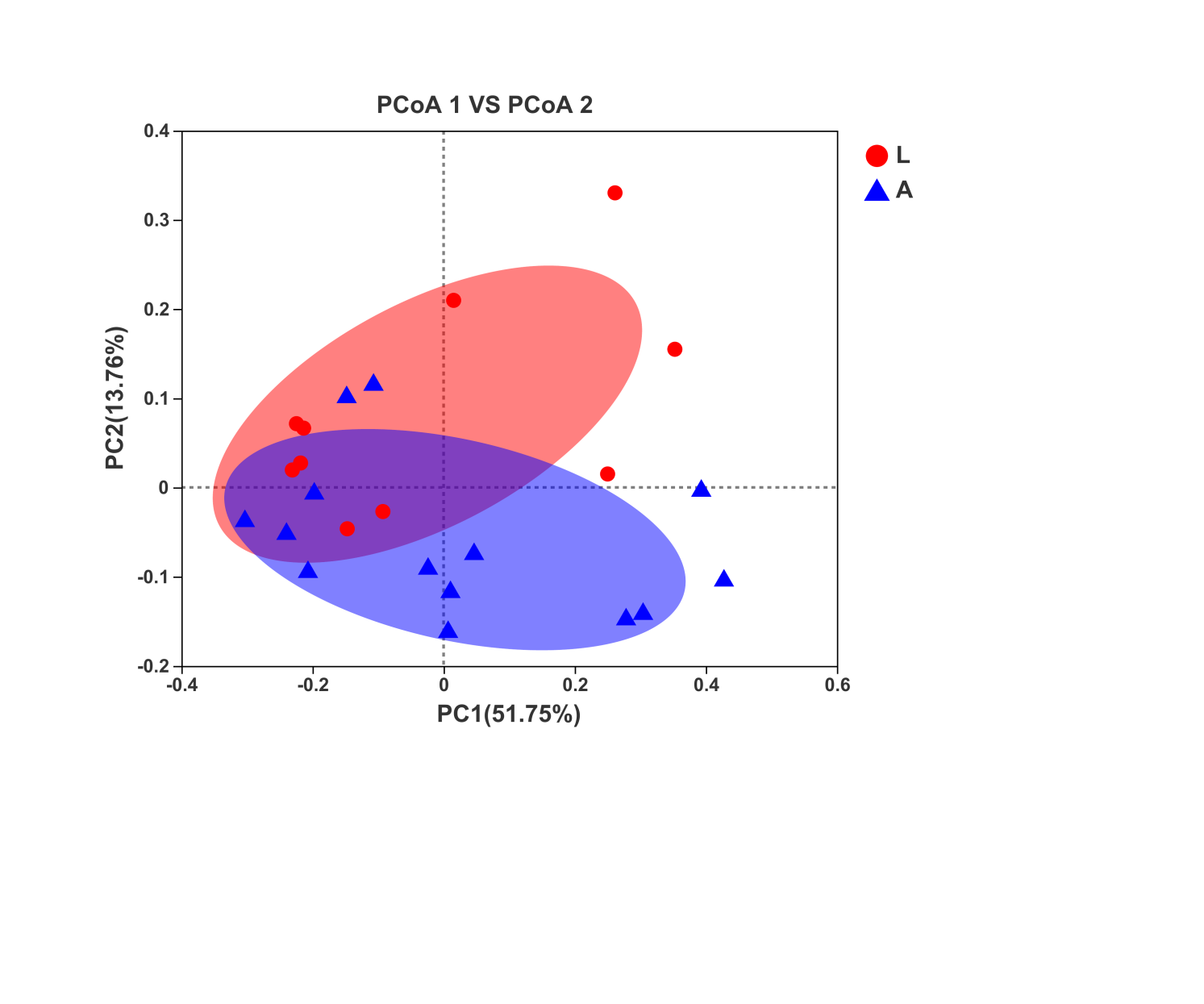


**Supplementary Figure 2.** Weighted UniFrac distance analysis of principal coordinates of gut microbiome differences between lactating and non-lactating bats.

**
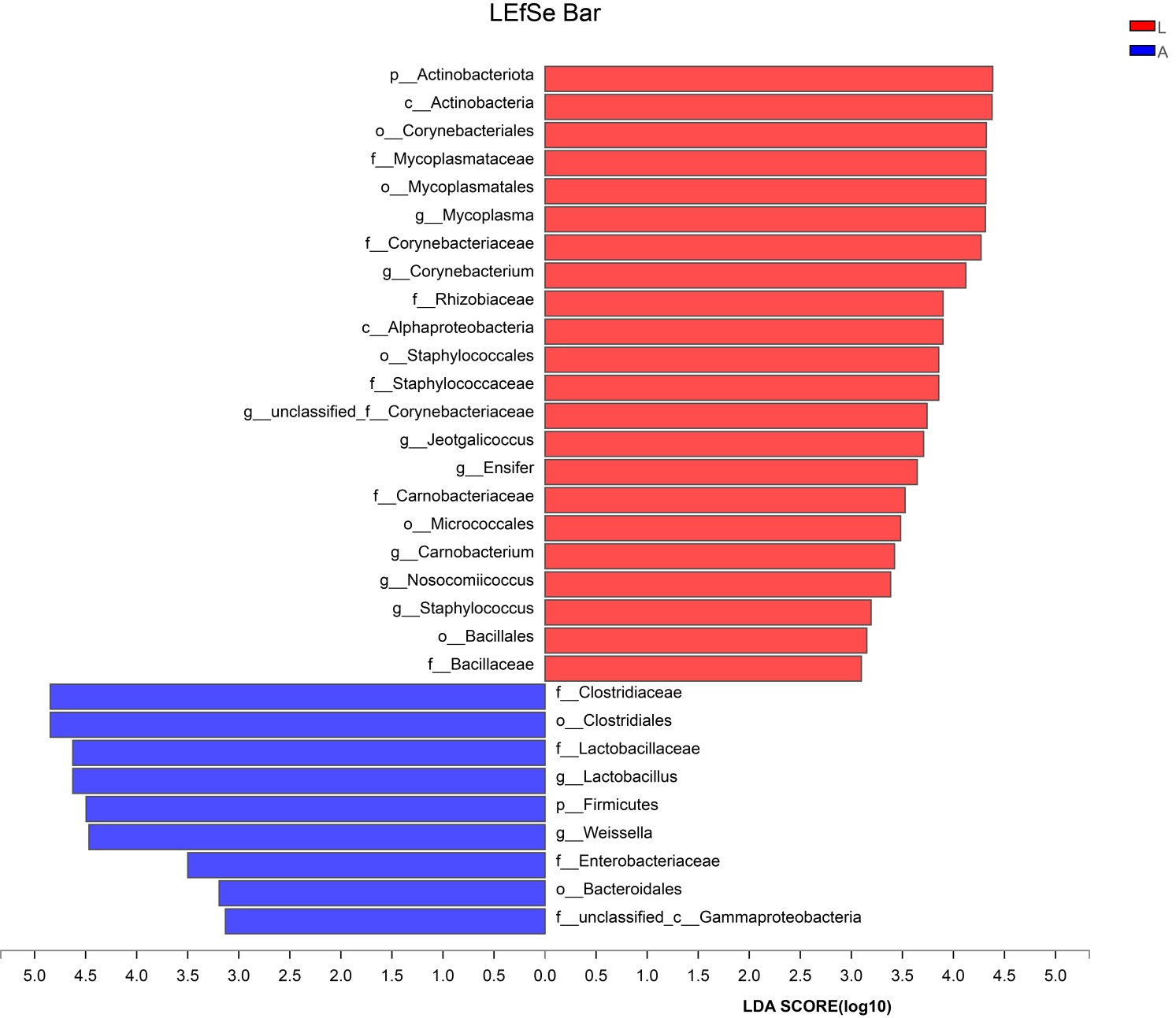
**

**Supplementary Figure 3.** Taxonomic differences of gut microbiota between lactating and non-lactating bats. Lineages with an LDA value >2.5 are displayed. The diameter of each dot is proportional to its effect size.

## Supplementary Tables

**Supplementary Table 1**. Classification information of diet and intestinal microorganism OTUs.

|  |  | OTUs | Counts |
| --- | --- | --- | --- |
| Diet | Total |  | 276 |
|  |  | Lactation | 138 |
|  |  | Non-lactation | 177 |
|  | Shared |  | 39 |
|  | Different |  |  |
|  |  | Lactation | 99 |
|  |  | Non-lactation | 138 |
| Gut microbiome | Total |  | 995 |
|  |  | Lactation | 615 |
|  |  | Non-lactation | 719 |
|  | Shared |  | 339 |
|  | Different |  |  |
|  |  | Lactation | 276 |
|  |  | Non-lactation | 380 |

OTUs, operational taxonomic units.

**Supplementary Table 2** . A checklist of insect species composition differences.

|  | Species name | A-Mean(%) | L-Mean(%) | P.value |  |
| --- | --- | --- | --- | --- | --- |
| Order | Coleoptera | 14.52 | 7.28 | 0.03 | * |
|  | Orthoptera | 1.56 | 0 | 0.001 | ** |
| Family | Limoniidae | 7.11 | 57.7 | 0.0002 | *** |
|  | Carabidae | 8.25 | 0.08 | 0.009 | ** |
|  | Lasiocampidae | 0 | 5.00 | 0.00002 | *** |
|  | Limacodidae | 0 | 3.43 | 0.04 | * |
| Genus | Rhipidia | 0.00 | 31.7 | 0.000009 | *** |
|  | Symplecta | 1.20 | 15.8 | 0.0002 | *** |
|  | Dicranomyia | 5.91 | 9.22 | 0.018 | * |
|  | unclassified_f__Psychodidae | 12.7 | 0.01 | 0.02 | * |
|  | Amara | 4.69 | 0.00 | 0.01 | * |
|  | Dendrolimus | 0.00 | 4.25 | 0.00003 | *** |
|  | Hylaea_f__Geometridae | 1.29 | 0.00 | 0.43 | * |

Mean (%) is the average relative abundance of species in different groups (* 0.01 < P ≤ 0.05, ** 0.001 < P ≤ 0.01, *** P ≤ 0.001).

**Supplementary Table 3.** Gut microbial composition difference test sheet.

|  | Species name | A-Mean(%) | L-Mean(%) | P.value |  |
| --- | --- | --- | --- | --- | --- |
| Phylum | Firmicutes | 95.86 | 89.34 | 0.01 | * |
|  | Actinobacteria | 1.05 | 5.77 | 0.004 | ** |
| Family | Lactobacillaceae | 6.95 | 0.10 | 0.001 | ** |
|  | Corynebacteriaceae | 0.20 | 3.71 | 0.0008 | *** |
|  | Mycoplasmataceae | 0.15 | 3.78 | 0.001 | ** |
|  | Staphylococcaceae | 0.46 | 2.03 | 0.001 | ** |
|  | Rhizobiaceae | 0.05 | 1.64 | 0.02 | * |
| Genus | Weissella | 7.77 | 0.30 | 0.03 | * |
|  | Lactobacillus | 6.95 | 0.092 | 0.001 | ** |
|  | Mycoplasma | 0.14 | 3.74 | 0.001 | ** |
|  | Corynebacterium | 0.16 | 2.56 | 0.05 | * |
|  | Jeotgalicoccus | 0.10 | 1.15 | 0.0004 | *** |
|  | unclassified_f__Corynebacteriaceae | 0.04 | 1.16 | 0.0001 | *** |

Mean (%) is the average relative abundance of species in different groups (* 0.01 < P ≤ 0.05, ** 0.001 < P ≤ 0.01, *** P ≤ 0.001).

**Supplementary Table 4.** Correlations between the gut microbial datasets and the dietary datasets summarized at different taxonomic levels

| Microbiome | Diet | R^2^ | P.value |
| --- | --- | --- | --- |
| OTU | OTU | 055 | 0.001 |
| OTU | Genus | 0.45 | 0.004 |
| OTU | Family | 0.32 | 0.02 |
| Genus | OTU | 0.30 | 0.01 |
| Genus | Genus | 0.17 | 0.11 |
| Genus | Family | 0.23 | 0.01 |
| Family | OUT | 0.34 | 0.01 |
| Family | Genus | 0.21 | 0.08 |
| Family | Family | 0.27 | 0.02 |

**Supplementary Table 5.** rocrustean correlations of the gut microbiome and dietary, both summarized at different taxonomic levels. The abscissa represents the classification of gut microbiome.

|  |  | OTU | Genus | Family |
| --- | --- | --- | --- | --- |
| lactation | M^2^ | 0.143 | 0.081 | 0.083 |
|  | P.value | 0.029 | 0.033 | 0.032 |
| non-lactation | M^2^ | 0.226 | 0.203 | 0.207 |
|  | P.value | 0.031 | 0.05 | 0.061 |

M^2^: Evaluate the correlation between the two ranking results.
